# Performance of DHI Score as a Predictor of Benign Paroxysmal Positional Vertigo in Geriatric Patients with Dizziness/Vertigo: A Cross-Sectional Study

**DOI:** 10.1371/journal.pone.0058106

**Published:** 2013-03-05

**Authors:** Amrish Saxena, Manish Chandra Prabhakar

**Affiliations:** Department of Medicine, Mahatma Gandhi Institute of Medical Sciences, Wardha, Maharashtra, India; Universidad Europea de Madrid, Spain

## Abstract

**Background:**

Dizziness/vertigo is one of the most common complaint and handicapping condition among patients aged 65 years and older (Geriatric patients). This study was conducted to assess the impact of dizziness/vertigo on the quality of life in the geriatric patients attending a geriatric outpatient clinic.

**Settings and Design:**

A cross-sectional study was performed in a geriatric outpatient clinic of a rural teaching tertiary care hospital in central India.

**Materials and Methods:**

In all consecutive geriatric patients with dizziness/vertigo attending geriatric outpatient clinic, DHI questionnaire was applied to assess the impact of dizziness/vertigo and dizziness associated handicap in the three areas of a patients’ life: physical, functional and emotional domain. Later, each patient was evaluated and underwent Dix-Hallpike maneuver by the physician who was blind of the DHI scoring of the patient.

**Statistical Analysis Used:**

We compared means and proportions of variables across two categories of benign paroxysmal positional vertigo (BPPV) and non-BPPV. For these comparisons we used Student’s *t*-test to test for continuous variables, chi-square test for categorical variables and Fisher’s exact test in the case of small cell sizes (expected value<5).

**Results:**

The magnitude of dizziness/vertigo was 3%. Of the 88 dizziness/vertigo patients, 19 (22%) and 69(78%) cases, respectively, were attributed to BPPV and non-BPPV group. The association of DHI score ≥50 with the BPPV was found to be statistically significant with x^2^ value = 58.2 at P<0.01.

**Conclusion:**

DHI Score is a useful tool for the prediction of benign paroxysmal positional vertigo. Correct diagnosis of BPPV is 16 times greater if the DHI Score is greater than or equal to 50. The physical, functional and emotional investigation of dizziness, through the DHI, has demonstrated to be a valuable and useful instrument in the clinical routine.

## Introduction

Dizziness or vertigo is an ill defined subjective sensation of postural instability or of illusory motion of self or environment either as a sensation of spinning or falling. Dizziness is one of the most common complaint among all outpatients and the single most common complaint, handicapping condition among patients aged 65 years and older. The prevalence of dizziness ranges from 4% to 30% in this age group [1], [2]. The lifetime prevalence of dizziness was 29.3%; the 1-year prevalence was found to be 18.2% in a community elderly population [3]. The annual prevalence of dizziness/vertigo in the adult population of 22.9% was reported in Germany [4]. Dizziness is associated with functional disability, and 10–20% of sufferers fall because of their symptoms [5], [6].

Dizziness/vertigo can be produced by peripheral vestibular disorders or central nervous system disorders or by combined lesions, and other conditions. Peripheral vestibular disorders include BPPV (benign paroxysmal positional vertigo), acute labyrinthitis, acute vestibular neuritis, Meniere’s disease, recurrent vestibulopathy, middle ear disease, otosclerosis and perilymphatic fistula. Among these, BPPV was the most frequent peripheral vestibular diagnosis reported in previous studies [4], [7], [8]. Posterior canal BPPV is more common than lateral canal and anterior canal BPPV, constituting approximately 85 to 95 percent of BPPV cases [9], [10]. The recurrence rate of BPPV is 27%, and relapse mainly occurs in the first few months [11]. Central nervous system disorders include cerebrovascular diseases such as transient ischaemic attacks (TIAs), brain stem disease, cerebellar disease, demyelinating diseases like multiple sclerosis, cerebellopontine angle tumor, posterior fossa lesions, infections and migraine. Other conditions which may cause dizziness are cervical spondylosis, postural hypotension, endocrinal diseases, drugs, vasculitis, anemia, polycythemia, psychological factors and cardiovascular diseases [2], [12].

The physical, emotional and functional disturbances that are associated with several kinds of dizzinesses harm the professional, social and domestic activities of the patient. It may cause difficulties on the patients’ daily life and even reduce their life quality. There is a paucity of literature about the personal and health care burden of dizziness and vertigo in the geriatric population. Little is known from the previous studies about the handicapping effect of dizziness imposed by vestibular causes in comparison to non-vestibular causes in the geriatric age group. Our study was conducted to assess the magnitude and impact of dizziness/vertigo on the quality of life in the geriatric patients attending a geriatric outpatient clinic in a rural medical college hospital. We also aimed to determine whether performance of Dizziness Handicap inventory (DHI) score would predict the diagnosis of benign paroxysmal positional vertigo (BPPV).

## Materials and Methods

### Ethics

The study was approved by the ethics committee of Mahatma Gandhi Institute of Medical Sciences (IRB00003623). We obtained a written informed consent from all study participants before enrolling them in the study.

### Setting

The study was conducted in the department of Medicine, Mahatma Gandhi Institute of Medical Sciences, Sevagram which is a 650-bedded teaching tertiary care hospital located in a town in central India. The department of Medicine has a daily adult outpatients (13–64 yrs) of approximately 200 and a geriatric outpatients (65 yrs or above) of about 30. Most patients visiting the hospital come from rural areas. All patients aged 65 years or above (geriatric patients) are screened and examined first in the geriatric outpatient clinic where after a brief history and examination, appropriate referrals to specialists are advised.

### Study Design

Between April 1, 2010 and July 31, 2010, a cross-sectional study was conducted to identify and assess geriatric patients with dizziness/vertigo attending geriatric outpatient clinic. All consecutive geriatric patients complaining of more than five episodes of dizziness/vertigo were included in the study. Patients were excluded if they had history of dementia, Parkinson’s disease, Alzheimer’s disease, severe depression, advanced malignancy or not provide written informed consent.

A diagnosis of benign paroxysmal positional vertigo (BPPV) required combination of a positive Dix-Hallpike test and a history of rotational vertigo, or positional vertigo, or recurrent dizziness with nausea and either imbalance or oscillopsia. The patients with vertigo/giddiness in which a characteristic torsional (rotational) nystagmus occurred after a brief latent period (1–5 seconds), lasting less than one minute, elicited during the Dix-Hallpike maneuver were considered to have a positive Dix-Hallpike test, while those without these features were considered to have a negative result [13]. Visualization of the nystagmus was aided by the use of optical Frenzel glasses, which eliminate visual fixation. The Dix-Hallpike maneuver is considered the gold standard test for the diagnosis of BPPV [14], [15]. Rotational vertigo was defined as an illusion of self-motion or object motion, and positional vertigo was defined as vertigo or dizziness precipitated by changes in head position such as lying down or turning in bed [4], [7], [16]. Patients with dizziness/vertigo who did not fulfill criteria for BPPV were classified in non-BPPV group.

Demographic data, including age and sex; personal habits like smoking; past history of hypertension, diabetes mellitus, ischemic heart disease or stroke; history of associated complaints like tinnitus, impaired hearing, pain in ear and ear discharge were obtained during baseline interview. Current smokers are classified as persons who reported smoking at least 100 cigarettes in their life and who currently smoke every day or on some days [17]. Presence of tinnitus was assessed by a positive response to the question: ‘Have you experienced any prolonged ringing, buzzing or other sound in your ears or head within the past one month…that is, lasting for 5 min or longer?’ Patients were classified as ***hypertensive*** if the mean of 2 blood pressure recordings was 140 mm Hg or greater for systolic and 90 mm Hg or greater for diastolic or if they reported having hypertension or the use of antihypertensive medications [18]. Patients with a systolic blood pressure between 120 to 139 mm Hg or a diastolic pressure between 80 to 89 mm Hg were classified as pre-hypertensive [18]. The patients were classified as ***diabetic*** if they reported having diabetes mellitus with medications even if their blood sugar were within the normal range. Some patients with fasting plasma glucose >126 mg/dl or 2-hour postprandial plasma glucose >200 mg/dl and with no past relevant history of diabetes mellitus were also classified as newly detected diabetics [19]. Patients with past documented history of myocardial infarction or angina were classified as having ***ischemic heart disease***. Patients with a self reported history or documentation of a ***cerebrovascular accident(s)*** (stroke) were also recorded.

Blood pressure and heart rate were measured after the participants had rested supine for at least 5 minutes. A standard sphygmomanometer with an appropriate-sized cuff was used. After blood pressure and heart rate were measured, the participant was helped into a standing position. His or her arm was placed on a portable stand that was level with the heart, and blood pressure and heart rate measurements were repeated immediately after standing and after 2 minutes. Postural or orthostatic hypotension was diagnosed when there was a drop in systolic blood pressure of 20 mm Hg or more or of 10 mm Hg in diastolic blood pressure two minutes after standing from the supine position with associated symptoms [20].

The Dizziness Handicap Inventory (DHI) Questionnaire, which has been developed by Jacobson and Newman (1990) was used to assess the impact of dizziness on patient’s daily life [21]. It evaluates the dizziness associated to the incapacities and handicap in the three areas of a patient’s life: Physical, functional and emotional (see Text S1). In the geriatric outpatient clinic, patients were asked to complete the Dizziness Handicap Inventory (DHI) consisting of 25 questions, and a total score (0–100 points) is obtained by summing ordinal scale responses, higher scores indicating more severe handicap. The 25 items were grouped into three dimensions: functional, emotional, and physical aspects of dizziness and unsteadiness. There were nine questions within each of the functional and emotional dimensions; with a maximum score of 4 for each item. Within the physical dimension there were seven questions with a maximum score of 4 for each. First, DHI questionnaire of the patient was filled by a social worker in the geriatric outpatient clinic and later the patient was evaluated and underwent Dix-Hallpike maneuver by the physician who was blind of the DHI scoring of the patient. After history and examination patients were referred to ENT outpatient clinic for specialist opinion and pure tone audiometry. Pure-tone audiometry was performed by audiologist in sound-treated booths, using standard ELKON 3N3 Multi clinical audiometer (India made). Hearing impairment was determined as the pure-tone average of audiometric hearing thresholds at 500, 1000 and 2000 Hz (PTA0.5–2 KHz), defining any hearing loss as PTA0.5–2 KHz >25 dB with the criterion based on the classification of the WHO [22].

### Statistical Analysis

We used SPSS software (version 16.0) to analyze the characteristics of the study population. We compared means and proportions of variables across two categories of BPPV and non-BPPV. For these comparisons we used the Student’s *t*-test for continuous variables, the chi-square test for categorical variables and the Fisher’s exact test in the case of small cell sizes (expected value<5). All comparisons were two-tailed. We considered the difference to be statistically significant if the P value was less than 0.05. To determine the best cut-off value of the DHI score for the diagnosis of BPPV, we used the receiver operating characteristic (ROC) curve analysis.

## Results

The overall magnitude of dizziness/vertigo was 3% (88 of 2900 geriatric patients screened in the geriatric OPD had dizziness/vertigo). Of the 88 dizziness/vertigo patients, 19 (22%) and 69(78%) cases, respectively, were assigned to the BPPV and non-BPPV group.

The mean age of patients in the BPPV group and non-BPPV group were 70 years (SD = 4.95) and 69 years (SD = 4.21) respectively. There was no significant difference between the mean age of patients in two categories (p>0.05).

Of the total 88 patients with dizziness/vertigo, 48 (55%) were males and 40 (45%) were females. There were 12 (63%) females in the BPPV group (n = 19) and 28 (41%) females were in the non-BPPV group (n = 69). There were 7 (37%) males in the BPPV group (n = 19) and 41 (59%) males in the non-BPPV group (n = 69). Although there were more females (63%) in the BPPV group and more males (59%) in the non-BPPV group, the difference was not significant (P>0.05) (Table 1).

**Table 1:** Sex distribution of geriatric patients among peripheral-vestibular vertigo and non-vestibular group.

sex	Peripheral-vestibular vertigo(n = 19)	Non-vestibular vertigo (n = 69)	Total (n = 88)
Male	7 (37%)	41 (59%)	48 (55%)
Female	12 (63%)	28 (41%)	40 (45%)

X^2^ = 3.06, P value >0.05. Values are number of patients. Figures in parenthesis represent the column percentage.

Of the 88 patients, 28 (32%) patients had hypertension, 12 (14%) patients had diabetes mellitus, 10 (11%) patients had ischemic heart disease, 5 (6%) patients had cerebrovascular stroke. The association of each comorbid illness i.e. hypertension, diabetes mellitus, ischemic heart disease and cerebrovascular stroke (cardiovascular diseases) with either group (BPPV vs. non-BPPV) was not found to be statistically significant (P>0.05). After classifying the patients in either group (BPPV vs. non-BPPV) as those with cardiovascular diseases (HT+DM+IHD+CV stroke) and those without cardiovascular diseases, we found no statistically significant association of cardiovascular diseases with BPPV/non-BPPV (P>0.05). Of the 88 vertigo patients, 14 (16%) patients were current smokers. There were 3 (21%) current smokers in the BPPV group and 11 (79%) current smokers in the non-BPPV group. The association of current smoking with either group (BPPV vs. non-BPPV) was not statistically significant (P>0.05)(Table 2). None of the patients in the BPPV group had postural hypotension, whereas 8 (9%) patients in the non-BPPV group had postural hypotension.

**Table 2:** Distribution of comorbid diseases/cardiovascular risk factors among peripheral-vestibular vertigo and non-vestibular vertigo group.

Comorbid diseases/cardiovascularrisk factors	Peripheral-vestibularvertigo (n = 19)	Non-vestibular vertigo (n = 69)	Total (n = 88)	P value
Hypertension*	5 (18%)	23 (82%)	28 (32%)	>0.05
Pre-hypertension*	8 (24%)	25 (76%)	33 (38%)	>0.05
Diabetes mellitus*	3 (25%)	9 (75%)	12 (14%)	>0.05
Ischemic heart disease*	2 (20%)	8 (80%)	10 (11%)	>0.05
Cerebrovascular stroke*	0 (0%)	5 (100%)	5 (6%)	>0.05
Current smokers*	3 (21%)	11 (79%)	14 (16%)	>0.05

* Expected cell count in one of the cells was less than 5 so Fisher’s exact test was used. Values are number of patients. Figures in parenthesis represent the row percentage.

ENT symptoms: Of the total 88 patients, 16 (18%) had tinnitus, 15 (17%) had impaired hearing, 8 (9%) had ear pain and 4 (4%) had ear discharge. The association of tinnitus with the BPPV group was found to be statistically significant (P<0.01) but impaired hearing, pain in ear and ear discharge were not significantly associated with either group i.e. BPPV vs. non-BPPV (P>0.05) (Table 3).

**Table 3:** Distribution of ENT Symptoms among peripheral-vestibular vertigo and non-vestibular vertigo group.

ENT symptoms	Peripheral-vestibular vertigo (n = 19)	Non-vestibular vertigo (n = 69)	Total (n = 88)	P value
Tinnitus*	12 (75%)	4 (25%)	16 (18%)	<0.01
Impaired hearing*	3 (20%)	12 (80%)	15 (17%)	>0.05
Ear pain*	2 (25%)	6 (75%)	8 (9%)	>0.05
Ear discharge*	0 (0%)	4 (100%)	4 (4%)	>0.05

* Expected cell count in one of the cells was less than 5 so Fisher’s exact test was used. Values are number of patients. Figures in parenthesis represent the row percentage.

**Figure 1:**
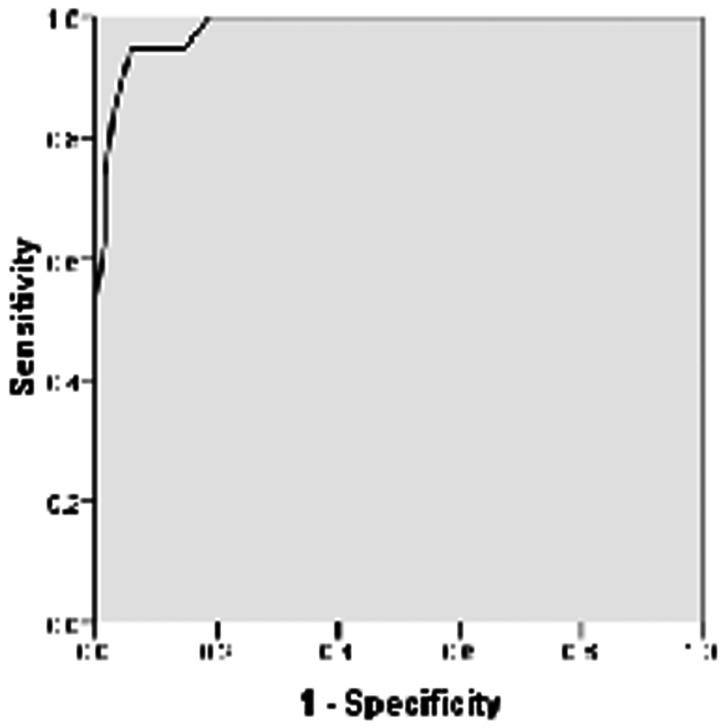
Receiver operating characteristic (ROC) curve for DHI scoring.

The mean DHI score of patients in the BPPV group was 65.68±11.41, whereas in the non-BPPV group it was 27.65±12.07. From the receiver operating characteristic (ROC) curve analysis (Figure 1), best cut-off of DHI score for diagnosis of BPPV was found to be 50 with area under the ROC curve (AUC) being 0.982, sensitivity of 94.7%, specificity of 94.2%, and positive likelihood ratio of 16.33 (Table 4). The association of DHI score ≥50 with the benign paroxysmal positional vertigo was found to be statistically significant with x^2^ value = 58.2 at P<0.01 (Table 5).

**Table 4:** Diagnostic and predictive accuracy of DHI score using Dix-Hallpike test as a gold standard.

Diagnostic/predictive accuracy	value
Sensitivity (%)	94.7
Specificity (%)	94.2
Positive predictive value (%)	81.0
Negative predictive value (%)	98.0
Positive likelihood ratio	16.33

**Table 5:** DHI score and Dix-Hallpike test.

	Dix-Hallpike test as a gold standard	
DHI score	Peripheral-vestibular vertigo	Non-vestibular vertigo
Score>/ = 50	18	4
Score<50	1	65

(X^2^ = 58.2 at P<0.01).

## Discussion

In our study, we were able to establish that a DHI score greater than or equal to 50 in a geriatric patient with dizziness/vertigo is a good predictor of benign paroxysmal positional vertigo. Patients in the BPPV group (mean DHI, 65.68±11.41) demonstrated greater handicap than those in the non-BPPV group (mean DHI, 27.65±12.07). The magnitude of dizziness/vertigo in our geriatric patients attending geriatric outpatient clinic was found to be 3%, which inflict a considerable health care burden.

Although there were more females (63%) in the BPPV group and more males (59%) in the non-BPPV group in our study, but the association of a particular gender with either group (BPPV vs. non-BPPV) was not found to be statistically significant. In previous studies, an association of vestibular vertigo with female sex was found in general population [7], [8]. The authors suggested that premenstrual or oral contraceptive-related hormonal changes may increase the risk of vestibular disorders.

The association of each comorbid illness i.e. hypertension, diabetes mellitus, ischemic heart disease or cerebrovascular stroke (cardiovascular diseases) with either group (BPPV vs. non-BPPV) was not found to be statistically significant. However in the past studies, hypertension and stroke had an independent effect on BPPV whereas diabetes and coronary heart disease was not associated with BPPV [7], [8]. The authors suggested that hypertension can lead to vascular damage to the inner ear and thus to BPPV. Vertebrobasilar ischemia was suggested as a predisposing factor for BPPV. It can be a sequel to labyrinthine ischemia that probably facilitates detachment of otoconia from the otolith membrane. Similarly, another study found an association between rotatory vertigo and stroke [23]. In a previous study done by Cohen et. al., diabetes was found to be unusually prevalent in BPPV patients [24]. In our study, if our sample size has been large with more number of patients with HT/DM/IHD/CV stroke in each subgroup, probably we could get a significant association of these cardiovascular risk factors with either group (BPPV vs. non-BPPV).

After classifying the patients in either group (BPPV vs. non-BPPV) as patients with cardiovascular diseases (HT+DM+IHD+CV stroke) and without cardiovascular diseases, we found no statistically significant association of these groups with BPPV/non-BPPV. If our study was done with a larger sample size having more number of patients in the cardiovascular group then a possible significant association between cardiovascular disease’ group and BPPV/non-BPPV group could be found. In the previous studies, an association was found between cardiovascular risk factors and dizziness [3], [5], [25]. The authors postulated that vascular disease seems important in the pathogenesis of dizziness in old age.

The association of current smoking with either group (BPPV vs. non-BPPV) was not statistically significant which is in line with previous studies [5], [7], [8]. In our study, 8 (9%) patients in the non-BPPV group had postural hypotension. Similarly, postural hypotension were noted in 14% of subjects in a previous study done by Ensrud et al. [20].

In our study, the association of tinnitus with the BPPV group was found to be statistically significant (P<0.01), reflecting the frequent otogenic origin of BPPV but impaired hearing, pain in ear and ear discharge were not significantly associated with either group i.e. BPPV vs. non-BPPV which is in line with the previous studies [5], [7], [26].

Our study has several strengths. Our patients represent typical rural Indian patients form central India. By including every consecutive outpatient we avoided the selection bias. We made a blind assessment (filling DHI questionnaire and physician evaluation) of the patient. We used a validated tool which can be used easily in outpatient clinic or bedside.

The limitation of our study is that the results of the study cannot be generalized to the community settings. Furthermore, no study patient underwent posturography, electronystagmography, tilt-table testing, caloric testing or neuroimaging (MRI) [27], [28], [29]. Although these tests may have helped to identify the presence of specific causes in a subset of our patients, but that was not the purpose of our study. Our risk factor analysis is limited by the cross-sectional design of the study. In patients with vertigo/dizziness having a positive Dix-Hallpike test, there might be few patients who had more than one factor involved (superimposed on a possible BPPV) in addition to the effects of aging itself.

### Conclusion

We conclude from the present study that DHI score is a useful tool for the prediction of benign paroxysmal positional vertigo (BPPV). Correct diagnosis of BPPV is 16 times greater if the DHI score is greater than or equal to 50. The physical, functional and emotional investigation of dizziness, through the DHI, has demonstrated to be a valuable and useful instrument in the clinical routine.

### Suggestion

Among patients presenting with dizziness/vertigo in geriatric outpatient clinic, the performance of DHI score in complement to careful clinical history could be one of the useful and valuable parameters for identifying patients with BPPV. Such patients can be subjected to therapeutic maneuvers (vestibular rehabilitation therapy), having more acceptances to this treatment, after realizing their own difficulties during the questionnaire application. It can avoid unnecessary and costly investigations especially in the resource-limited settings. It is simple and must be included in routine evaluation of geriatric patients with dizziness/vertigo at daily dizziness clinic.

## Supporting Information

Text S1 **DHI Questionnaire.**
(PDF)
