# Supplementary material for: Performance of DHI Score as a Predictor of Benign Paroxysmal Positional Vertigo in Geriatric Patients with Dizziness/Vertigo: A Cross-Sectional Study
Source: PLoS One. 2013 Mar 5;8(3):e58106. doi: 10.1371/journal.pone.0058106 (PMC3589349; doi:10.1371/journal.pone.0058106)
Supplement: Text S1 — DHI Questionnaire. (PDF) [file pone.0058106.s001.pdf]

NAME: ____________________ DATE: ______ AGE: ______ Score Totals: ____; E____; F____; P____
(100) (36) (36) (28)

DIZZINESS HANDICAP INVENTORY

Please **CIRCLE** the correct response:

1. I have dizziness/unsteadiness: [*1*] 1 per month [*2*] > 1 but < 4 per month [*3*] more than one per week
2. My dizziness/unsteadiness is: [*1*] mild [*2*] moderate [*3*] severe

INSTRUCTIONS: (Please read carefully): The purpose of the scale is to identify difficulties that you may be experiencing because of your dizziness or unsteadiness. Please answer "YES", "SOMETIMES", or "NO" to each question. **Answer each question as it pertains to your dizziness or unsteadiness problem only.**

	YES	SOMETIMES	NO
P1. Does looking up increase your problem?	☐	☐	☐
E2. Because of your problem, do you feel frustrated?	☐	☐	☐
F3. Because of your problem, do you restrict your travel for business or recreation?	☐	☐	☐
P4. Does walking down the aisle of a supermarket increase your problem?	☐	☐	☐
F5. Because of your problem, do you have difficulty getting into or out of bed?	☐	☐	☐
F6. Does your problem significantly restrict your participation in social activities such as going out to dinner, going to movies, dancing, or to parties?	☐	☐	☐
F7. Because of your problem, do you have difficulty reading?	☐	☐	☐
P8. Does performing more ambitious activities like sports, dancing, household chores such as sweeping or putting dishes away increase your problem?	☐	☐	☐
E9. Because of your problem, are you afraid to leave your home without someone accompanying you?	☐	☐	☐
E10. Because of your problem, have you been embarrassed in front of others?	☐	☐	☐
P11. Do quick movements of your head increase your problem?	☐	☐	☐
F12. Because of your problem, do you avoid heights?	☐	☐	☐
P13. Does turning over in bed increase your problem?	☐	☐	☐
F14. Because of your problem, is it difficult for you to do strenuous house work or yard work?	☐	☐	☐
E15. Because of your problem, are you afraid people may think you are intoxicated?	☐	☐	☐
F16. Because of your problem, is it difficult for you to go for a walk by yourself?	☐	☐	☐
P17. Does walking down a sidewalk increase your problem?	☐	☐	☐
E18. Because of your problem, is it difficult for you to concentrate?	☐	☐	☐
F19. Because of your problem, is it difficult for you to walk around your house in the dark?	☐	☐	☐
E20. Because of your problem, are you afraid to stay home alone?	☐	☐	☐
E21. Because of your problem, do you feel handicapped?	☐	☐	☐
E22. Has your problem placed stress on your relationships with members of your family or friends?	☐	☐	☐
E23. Because of your problem, are you depressed?	☐	☐	☐
F24. Does your problem interfere with your job or household responsibilities?	☐	☐	☐
P25. Does bending over increase your problem?	☐	☐	☐

Jacobson GP, Newman CW. The development of the Dizziness Handicap Inventory. Arch Otolaryngol Head Neck Surg. 1990 Apr; 116(4): 424-7

SCORING METHOD FOR DIZZINESS HANDICAP INVENTORY

E= Emotionally based questions (#'s 2, 9, 10, 15, 18, 20, 21, 22, 23)
9 questions total

F = Functionally based questions (#'s 3, 5, 6, 7, 12, 14, 16, 19, 24)
9 questions total

P = Physically based questions (#'s 1, 4, 8, 11, 13, 17, 25)
7 questions total

SCORE VALUES

"YES" =	4 POINTS
"SOMETIMES" =	2 POINTS
"NO" =	0 POINTS

FINAL SCORES

EMOTIONAL = total sum of columns for the "E" questions above / Pt's total (9X4=36) or,
Patients Score (E questions) / Pt total score

FUNCTIONAL = total sum of columns for the "F" questions above / total possible (9X4=36) or,
Patients Score (F questions) / Pt total score

PHYSICAL = total sum of columns for the "P" questions above / total possible (7X4=28) or,
Patients Score (F questions) / Pt total score

TOTAL COMPOSITE SCORE = (E + F + P questions) / 100

Jacobson GP, Newman CW. The development of the Dizziness Handicap Inventory. Arch Otolaryngol Head Neck Surg. 1990 Apr; 116(4): 424-7
